# Association of Chronic Kidney Disease With Plasma NfL and Other Biomarkers of Neurodegeneration: The H70 Birth Cohort Study in Gothenburg

**DOI:** 10.1212/WNL.0000000000207419

**Published:** 2023-07-18

**Authors:** Anna Dittrich, Nicholas J. Ashton, Henrik Zetterberg, Kaj Blennow, Anna Zettergren, Joel Simrén, Tobias Skillbäck, Sara Shams, Alejandra Machado, Eric Westman, Michael Schöll, Ingmar Skoog, Silke Kern

**Affiliations:** From the Neuropsychiatric Epidemiology Unit (A.D., A.Z., T.S., I.S., S.K.), Sahlgrenska Academy, University of Gothenburg; Department of Psychiatry Cognition and Old Age Psychiatry (A.D., I.S., S.K.), Sahlgrenska University Hospital, Mölndal; Department of Psychiatry and Neurochemistry (N.J.A., H.Z., K.B., J.S., M.S.), Institute of Neuroscience and Physiology, the Sahlgrenska Academy at the University of Gothenburg, Mölndal Campus, Sweden; King's College London (N.J.A.), Institute of Psychiatry, Psychology and Neuroscience, Maurice Wohl Institute Clinical Neuroscience Institute; NIHR Biomedical Research Centre for Mental Health and Biomedical Research Unit for Dementia at South London and Maudsley NHS Foundation (N.J.A.), London, UK; Centre for Age-Related Medicine (N.J.A.), Stavanger University Hospital, Norway; Clinical Neurochemistry Laboratory (H.Z., K.B., J.S.), Sahlgrenska University Hospital, Mölndal, Sweden; UK Dementia Research Institute at UCL (H.Z.); Department of Neurodegenerative Disease (H.Z., M.S.), UCL Institute of Neurology, London, UK; Hong Kong Center for Neurodegenerative Diseases (H.Z.), China; Care Sciences and Society (S.S.), Karolinska Institutet, and Department of Radiology; Department of Clinical Neuroscience (S.S.), Karolinska University Hospital, Stockholm, Sweden; Department of Radiology (S.S.), Stanford University, CA; Division of Clinical Geriatrics (A.M., E.W.), Department of Neurobiology, Care Sciences and Society (NVS), Karolinska University Hospital; Division of Insurance Medicine (A.M.), Department of Clinical Neuroscience, Karolinska Institutet, Stockholm, Sweden; Department of Neuroimaging (E.W.), Centre for Neuroimaging Sciences, Institute of Psychiatry, Psychology and Neuroscience, Kings College London, UK; and Wallenberg Center of Molecular and Translational Medicine (M.S.), University of Gothenburg, Sweden.

## Abstract

**Background and Objectives:**

Studies associate chronic kidney disease (CKD) with neurodegeneration. This study investigated the relationship between kidney function, blood, CSF, and structural brain MRI markers of neurodegeneration in a sample including individuals with and without CKD.

**Methods:**

Participants from the Gothenburg H70 Birth Cohort Study, with data on plasma neurofilament light (P-NfL), estimated glomerular filtration rate (eGFR), and structural brain MRI were included. Participants were invited to also have the CSF collected. The primary endpoint of this study was to determine any association between CKD and P-NfL. Secondary endpoints included cross-sectional associations between CKD, eGFR, and CSF-derived and MRI-derived markers of neurodegeneration and Alzheimer disease (AD) pathology (MRI: cortical thickness, hippocampal volume, lateral ventricle volume, and white matter lesion volume; CSF: β-amyloid (Aβ) 42, Aβ42/40, Aβ42/p-tau, t-tau, p-tau, and NfL). Participants with P-NfL and eGFR at baseline were re-examined on eGFR, 5.5 (5.3–6.1) years (median; IQR) after the first visit, and the predictive value of P-NfL levels on incident CKD was estimated longitudinally, using a Cox proportional hazards model.

**Results:**

We included 744 participants, 668 without CKD (age 71 [70–71] years, 50% males) and 76 with CKD (age 71 [70–71] years, 39% males). Biomarkers from the CSF were analyzed in 313 participants. A total of 558 individuals returned for a re-examination of eGFR (75% response rate, age 76 [76; 77] years, 48% males, 76 new cases of CKD). Participants with CKD had higher P-NfL levels than those with normal kidney function (median; 18.8 vs 14.1 pg/mL, *p* < 0.001), while MRI and CSF markers were similar between the groups. P-NfL was independently associated with CKD after adjustment for confounding variables, including hypertension and diabetes (OR; 3.231, *p* < 0.001), in a logistic regression model. eGFR and CSF Aβ 42/40: R = 0.23, *p* = 0.004 correlated in participants with Aβ42 pathology. P-NfL levels in the highest quartile were associated with incident CKD at follow-up (HR; 2.39 [1.21: 4.72]).

**Discussion:**

In a community-based cohort of 70-year olds, P-NfL was associated with both prevalent and incident CKD, while CSF and/or imaging measures did not differ by CKD status. Participants with CKD and dementia presented similar levels of P-NfL.

Decline in renal function and an increasing prevalence of neurodegenerative conditions, including Alzheimer disease (AD), cerebrovascular disease, and polyneuropathy, are all related with aging.^1,2^ Chronic kidney disease (CKD) has a prevalence of approximately 11% in the western world^3^ and is one of the fastest growing causes of death globally alongside dementia.^4^ Dementia had an estimated global prevalence of 57 million people in 2019, a number expected to increase to 153 million by 2050.^1^

CKD has previously been associated with blood-based biomarkers of neurodegeneration (phosphorylated tau [p-tau], amyloid beta 42/40(Aβ42/40), and neurofilament light protein [NfL])^5-9^ and different neurodegenerative conditions including dementia^10^ and polyneuropathy.^11^ This association has previously been examined in the clinical context of different patient groups, including patients from memory clinics.^12-14^ Because the kidney has a function in aminoacid recycling and clearance of circulating peptides, smaller proteins, such as insulin (5.8 kDa) and glucagon (3.5 kDa), pass the glomeruli pores and are long known to be cleared in the kidney tubuli to a significant degree.^15^ Larger proteins such as albumin (67 kDa) are not normally filtered out by the glomeruli, but can leak to the urine in a state of albuminuria.^16^ This occurs in a smaller fraction of patients with mild CKD, but is more frequent as the disease progresses.^17^ The rapid advances in the development of blood-based biomarkers of neurodegeneration also brings a need for understanding what comorbidities influence the measurements. Some authors suggest that CKD and kidney function could alter a biomarkers' normal reference range and should be considered when used in clinical screening and diagnosis in cognitively healthy populations or in clinical studies of neurodegeneration.^6,7,18,19^

Plasma neurofilament light protein (P-NfL) is a promising biomarker of neurodegeneration for use in a primary care setting because it is analyzed from blood and not CSF. P-NfL segregates depression from dementia in older individuals, and while only mildly elevated in patients with AD, it is an efficient marker for ruling out underlying neurodegeneration.^20,21^ P-NfL is also high in amyotrophic lateral sclerosis (ALS), atypical parkinsonian disorders, frontotemporal dementia, and in patients with Down syndrome with AD, providing evidence of an underlying neurodegenerative cause of a patient's symptoms.^21-23^

Studies on the influence of several comorbidities and different blood-based biomarkers have reported an influence of kidney function on P-NfL.^6,7,9^ However, studies specifically focused on the interaction between kidney function and different neurodegenerative markers in a community-based setting of older individuals with CKD and normal kidney function are currently few. The Alzheimer Association has specifically highlighted the need for studies on the influence of kidney disease on the diagnostic performance of P-NfL as one of the top research priorities in its recent statement paper on the use of blood-based biomarkers.^24^ The aim of this study was to investigate kidney function associations with P-NfL and several other markers of neurodegeneration in individuals with and without CKD.

## Methods

### Study Design and Population Using Data From the Gothenburg H70 Birth Cohort Study

**Figure 1:**
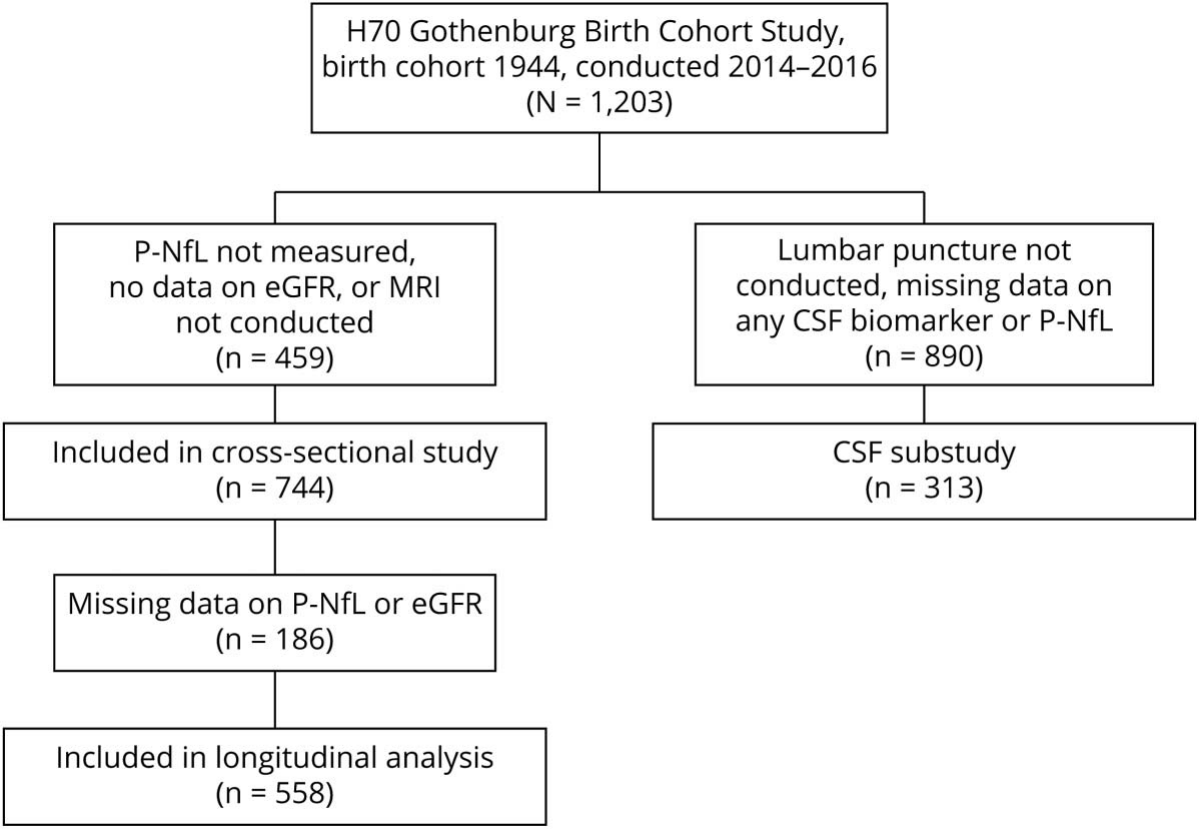
Flowchart of the Inclusion Process for the Study

This study was conducted in participants from the Gothenburg H70 Birth Cohort 1944^25^ with data available on P-NfL, estimated glomerular filtration rate (eGFR), and established structural MRI variables in studies of dementia, mean cortical thickness,^26^ mean lateral ventricle volume,^26^ mean hippocampal volume,^26^ and total white matter lesion volume^27^ (n = 744) (Figure 1). The cohort is derived from a population-based study that invited all citizens of Gothenburg, born on specific birth dates in 1944, to attend a health examination the year they turned 70 years of age. In total, 1,203 accepted the invitation (response rate 72.2%), and the examinations were conducted between 2014 and 2016, previously described in detail.^25^ The primary endpoint of this study was to determine any association between CKD and P-NfL. Secondary endpoints included associations between CKD and other markers of neurodegeneration and any association between kidney function measured as eGFR and CSF-derived and MRI-derived markers of neurodegeneration.

Besides health interviews, blood sampling, and physical examinations, all participants who did not present with any contraindications were also invited to a brain MRI examination and a lumbar puncture. Due to the limited participation rate for CSF sampling, associations determined with CSF biomarkers were conducted as a substudy of participants with available CSF and complete data on fluid-based biomarkers of neurodegeneration (n = 313). Blood samples were collected at the first study visit, brain MRI was conducted within 3 months of the initial study visit, and lumbar puncture was performed within 2 months of the MRI examination.

### Standard Protocol Approvals, Registrations, and Patient Consents

This study was conducted according to the Helsinki Declaration approved by the Regional Ethical Review Board in Gothenburg (869-13, T076-14, T166-14, 976-13, 127-14, T936-15, 006-14, T703-14, 006-14, T201-17, T915-14, 959-15, and T139-15). All the participants and/or their close relatives gave written consent before any study-related procedures were performed.

### Data Availability

Anonymized data can be obtained by reasonable request from any qualified investigator.

### Baseline Health Interview and Medical Examination

All participants attended a health examination at the Neuropsychiatric Clinic at Sahlgrenska University Hospital in Gothenburg, Sweden, conducted by the Gothenburg H70 Birth Cohort Study team. Participants underwent a health interview covering social and medical aspects. Anthropometric variables were determined, including weight, height, and blood pressure. BMI was calculated as weight (kg)/height (m^2^), eGFR was calculated according to CKD-epi,^28^ using the formula mentioned further. CKD was defined as an eGFR below 60 mL/min/1.73 m^2^.^29^
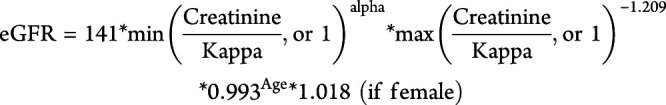


Kappa = 0.7 (females) or 0.9 (males)

Alpha = -0.329 (females) or -0.411 (males)

Medical comorbidities were determined through a combination of health interviews, data in the Swedish National In-patient register, and collected clinical variables. CDR was assessed by research nurses with specific training, and dementia was diagnosed according to the Diagnostic and Statistical Manual of Mental Disorders, 3rd edition, revised criteria. CDR score and dementia diagnoses were also verified by study physicians in consensus conferences. Hypertension was defined as a systolic blood pressure >140 mm Hg, a diastolic blood pressure >90 mm Hg, or a history of hypertension with ongoing medication reported by the participant.

A stroke was determined if the participant or a close relative reported the diagnosis, if it was diagnosed in the Swedish National In-patient register, or there were stroke-specific findings on the MRI scan. A history of transitory ischemic attacks was not classified as a stroke. Diabetes was defined as a previous diagnosis of diabetes or if the participant presented an fP-glucose ≥7.0 mmol/L at the study visit.

### Fluid-Based Biomarker Analysis

Blood samples were collected in the morning after overnight fasting. Creatinine, fasting plasma glucose (fP-glucose), homocysteine, C-reactive protein, and LDL cholesterol were analyzed at the Sahlgrenska Clinical Chemistry laboratory. P-NfL was analyzed using the NF-Light kit on a Simoa HD-X Analyzer (Quanterix, Billerica, MA) at the Neurochemistry Laboratory at Sahlgrenska University Hospital, Mölndal, according to the manufacturer's instructions. Quality control samples presented a 7.6% repeatability and 8% intermediate precision (at 6.6 pg/mL) and a 7.2% repeatability and 7.8% intermediate precision (at 50.5 pg/mL).

Blood was collected for *APOE* genotyping with the KASPar PCR SNP genotyping system (LGC Genomics, Hoddesdon, Herts, UK). *APOE* ε2, ε3, and ε4 alleles were defined by single-nucleotide variants rs7412 and rs429358 (n = 744).

Lumbar puncture was performed on a separate day by a medical doctor specialized in psychiatry or neurology.^25^ CSF samples were centrifuged, gently mixed, and stored at −80°C until analysis.

T-tau and p-tau in the CSF were analyzed using commercial enzyme-linked immunosorbent assays (ELISA) (INNOTEST htau Ag and PHOSPHO_TAU [181P], Fujirebio [formerly Innogenetics], Ghent, Belgium),^30,31^ while INNOTEST Aβ1-42 was used to measure the 42-amino acid long version of Aβ (Aβ42).^32^ The V-PLEX Aβ Peptide Panel 1 (6E10) Kit (MesoScale Discovery, Rockville, MD) was used to measure the CSF Aβ_42/40_ ratio.^33^ Aβ pathology status was defined as Aβ42 levels below 530 pg/mL in accordance with a previously conducted longitudinal study predicting incident AD.^34^

CSF NfL was measured with an ELISA developed at the Mölndal Clinical Neurochemistry Laboratory.^35,36^ All assays are used in routine clinical analyses at the Mölndal Clinical Neurochemistry laboratory.^37^ Analytical runs passed quality control criteria for the calibrators, and internal quality control samples were approved as previously described in detail.^37^

### MRI

Brain MRI was conducted at the Aleris Clinic in Gothenburg using a 3.0T Philips Achieva system as previously described in detail.^25^ The mean cortical thickness and mean lateral ventricular and hippocampal volumes were quantified using FreeSurfer 6.0.0 through the TheHiveDB,^38^ and the mean volume between the left and right sides was calculated for ventricles and hippocampus.^6^ Total white matter lesion volumes were measured using the open-source segmentation toolbox LST 2.0.15 implemented in the SPM software as previously described.^39^ All volumes were normalized by ratio to total intracranial volume, as previously described.^6^

### Follow-up Examination

Participants in H70 Birth Cohort 1944 were invited for a re-examination after the age of 75 years. Individuals without CKD and with P-NfL and eGFR measured at baseline were included in the study for a longitudinal evaluation on incident CKD, defined as a follow-up eGFR < 60 mL/min/1.73 m^2^.

### Statistical Methods

Categorical variables are presented as n (%) and continuous variables as median (interquartile range [IQR]) unless otherwise specified. Categorical variables were compared between groups with the χ^2^ or Fisher exact test as appropriate. For groupwise comparisons of continuous variables, the Mann-Whitney *U* test or Kruskal-Wallis test was used. Correlations were determined using Spearman correlations. For the cross-sectional logistic regression analysis with CKD as the dependent variable and P-NfL as the independent continuous variable, 3 models were constructed with age, sex, and years of education as covariates in model I. Model II included additional adjustments for medical history of hypertension or diabetes and model III with additional adjustments for smoking, BMI, fP-glucose, S-LDL cholesterol, P-CRP, and P-homocysteine. Non-normally distributed variables were log transformed before analysis. For P-NfL, 1 outlying sample (more than 10 SD higher than the mean) was identified, and statistical tests were performed with and without this individual as a sensitivity analysis. The analyses of the association between P-NfL and eGFR was repeated in the full H70 Birth Cohort of 1,151 individuals, with data on eGFR and P-NfL. Cross-sectional linear regression analysis was also performed with P-NfL as the independent continuous variable and eGFR as the dependent variable, adjusted for the same variables as in the logistic regression model. For longitudinal statistical analysis of the predictive value of P-NfL on incident CKD, a Cox proportional hazards model was used, adjusting for the same variables as in the other regression models. P-NfL divided by quartiles was used as a categorical variable in the Cox proportional hazards model. The proportional hazards assumption was tested through a hierarchical regression strategy, where each regression model was followed up by the addition of time-dependent interaction terms. No model was significantly improved through this addition. GraphPad Prism (version 9.0.0, GraphPad Software) was used for the plots and statistics therein, and SPSS (version 26, IBM) was used for all other statistical analyses.

## Results

### Characteristics

From the Gothenburg H70 Birth Cohort 1944 (N = 1,203, 559 men, 644 women, 96.5% born in Europe), 744 individuals met the inclusion criteria for this study. The characteristics of the participants are provided in Table 1, with additional information on medications as supplementary material (eTable 1, links.lww.com/WNL/C849) (n = 744). Seventy-six of the 744 individuals (10.2%) included in the study experienced CKD. Comparing participants with and without CKD, there were no statistically significant differences in sex distribution, age, and years of education (Table 1). Considering medical history, the prevalence of hypertension was higher in participants with CKD, as was their BMI. There was no difference in the distribution of CDR = 0 and 0.5 between participants with or without CKD, and the prevalence of dementia was overall low (<2%). The prevalence of *APOE* ε4 genotype, stroke, and diabetes and fasting levels of glucose did not differ between the 2 groups. By definition, eGFR was lower in participants with CKD, and creatinine levels were higher. As expected, the vitamin B deficiency marker homocysteine, the inflammatory marker CRP, and S-triglycerides were higher in participants with CKD, while B-Hb and S-HDL cholesterol levels were lower.^40,41^ P-NfL levels were also higher in participants with CKD (Table 1).

**Table 1:**
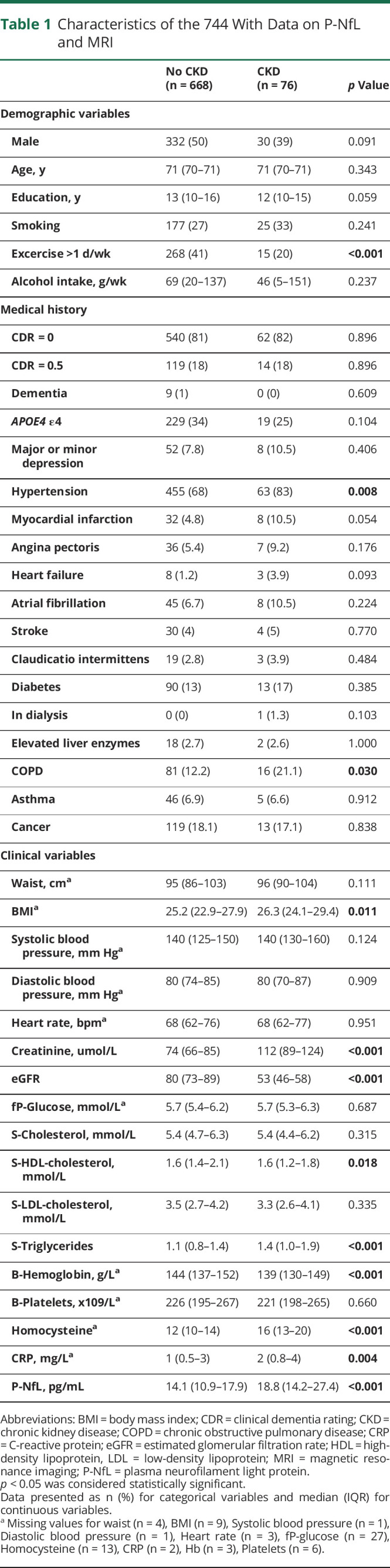
Characteristics of the 744 With Data on P-NfL and MRI

### Imaging-Based Measurements of Neurodegeneration

**Figure 2:**
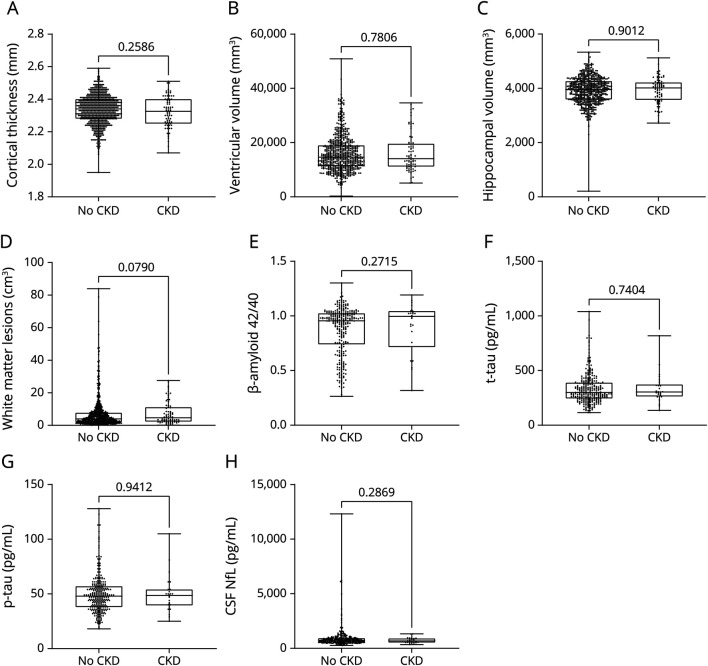
Structural MRI Measurements and CSF Biomarkers in Participants Without and With CKD Structural MRI measurements of cerebral regions related to neurodegeneration presented for participants without and with CKD (A–D). Mean cortical thickness (A), mean lateral ventricular volume (B), mean hippocampal volume (C), and total white matter lesion volume (D) were measured. CSF biomarkers related to neurodegeneration were also analyzed (E–H). Aβ42/40-ratio (E), t-tau (F), p-tau (G), and NfL (H) in CSF were measured in participants without and with CKD. n = 744 in panel A–D (No CKD = 668, CKD = 76) and n = 313 in panel E–H (No CKD = 287, CKD = 26). The Mann-Whitney *U*-test was used for groupwise comparisons. NfL = neurofilament light protein; p-tau = phosphorylated tau; t-tau = total tau.

Measurements of mean cortical thickness (Figure 2A, *p* = 0.2586), mean lateral ventricular volume (Figure 2B, *p =* 0.7806, mean hippocampal volume (Figure 2C, *p* = 0.9012), and total volume of white matter lesions (Figure 2D, *p* = 0.0790) were comparable between participants with and without CKD. One participant with dementia presented very small volumetric measurements. Exclusion of the participant did not change statistical outcomes in a sensitivity analysis and was therefore kept in the sample. Correlation analysis of eGFR and these markers of neurodegeneration did not reveal any significant correlations (eTable 2, inks.lww.com/WNL/C849).

### CSF-Based Measurements of Neurodegeneration

CSF concentrations of several different markers of neurodegeneration were analyzed in the 313 participants who accepted CSF sampling. Of them, 26 (8.3%) had CKD. Both groups presented levels of CSF Aβ42/40 (Figure 2E, *p* = 0.2715), t-tau (Figure 2F, *p* = 0.7404), p-tau (Figure 2G, *p* = 0.9412), and NfL (Figure 2H, *p* = 0.2869) in the same magnitude, comparing participants with or without CKD.

### Associations Between P-NfL and Kidney Function

**Figure 3:**
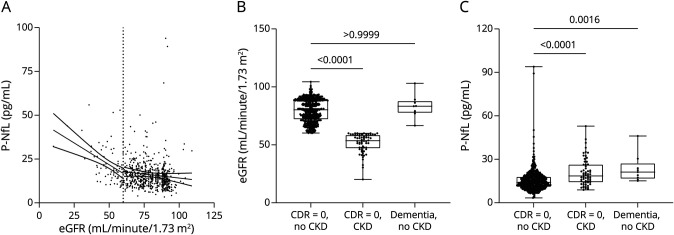
Associations Between P-NfL and Kidney Function, Stratified by CKD and Dementia Scatterplot of P-NfL and eGFR in the study participants (n = 743, 1 outlier was not included in the figure, but was included in the statistics, n = 744), with linear regression curves stratified by CKD status (no CKD, n = 668 and CKD, n = 76) (A). Kidney function measured as eGFR (B) and P-NfL levels (C) in participants without cognitive impairment (CDR = 0), stratified by CKD status (no CKD, n = 540 and CKD, n = 62), and in participants with dementia and normal kidney function (n = 9). Linear regression lines in figure A were performed on untransformed P-NfL values to allow for the presentation of clinically relevant data in the panel. Groupwise comparisons were performed with the Kruskal-Wallis test. eGFR = estimated glomerular filtration rate; NfL = neurofilament light protein; P = plasma.

P-NfL increased with lower kidney function measured as eGFR (Figure 3A). Stratified linear regressions revealed a steeper slope in participants with CKD (beta = −0.496, *p < 0.*001) compared with participants without CKD (beta = −0.091, *p < 0.*137, Figure 3A). To disentangle the potential confounding effects of CKD and dementia on the association between eGFR and P-NfL levels, these variables were compared in 3 distinct subgroups: participants with CDR = 0 and no CKD (n = 540), participants with CDR = 0 and CKD (n = 62), and participants with dementia, but no CKD (n = 9). Participants with CDR = 0 and participants with dementia, both without CKD, presented similar eGFR levels (*p* > 0.999) (Figure 3B), while P-NfL levels were higher in those with dementia (*p* = 0.0016). As expected, in participants with CKD and CDR = 0, eGFR was lower than in those without CKD (Figure 3B) (*p < 0.*001). P-NfL levels were also higher in participants with normal cognitive function and CKD compared with those without CKD (*p < 0.*001, both groups CDR = 0) (Figure 3C). In a sensitivity analysis, the results from these analyses were replicated in all 1,151 participants with data on eGFR and P-NfL in the full H70 Birth Cohort Study (eFigure 1, links.lww.com/WNL/C849).

### Logistic and Linear Regression Analyses Between CKD, eGFR, and P-NfL

A logistic regression analysis of the relationship between P-NfL and CKD, adjusted for age, sex, and education was performed (model I), demonstrating a statistically significant association between P-NfL and CKD (Table 2). Additional adjustments for other risk factors of CKD and neurodegeneration, including hypertension and diabetes (model II) and smoking, BMI, fasting plasma glucose, LDL cholesterol, CRP, and homocysteine (model III) did not alter the statistical significance (Table 2). Excluding participants with dementia (n = 9) did not alter the statistical significance of any model, although the OR was slightly higher (eTable 3, links.lww.com/WNL/C849). One individual without CKD presented with extreme levels of P-NfL, and the analyses were therefore repeated without this individual, showing slightly higher OR and similar *p* values (eTable 4). There was also a statistically significant association between P-NfL and eGFR, assessed using a linear regression models adjusted for the same confounders as the logistic regression (Table 2).

**Table 2:**
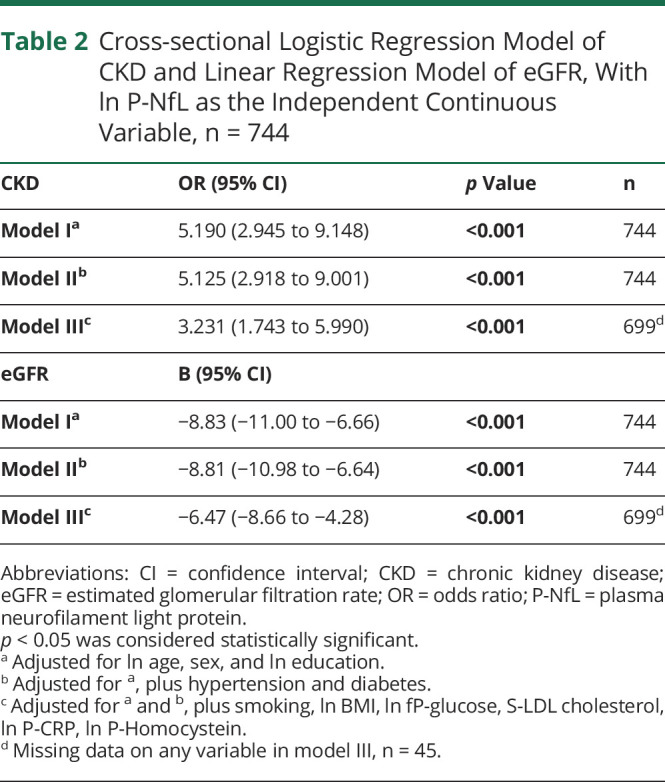
Cross-sectional Logistic Regression Model of CKD and Linear Regression Model of eGFR, With ln P-NfL as the Independent Continuous Variable, n = 744

### Cox Proportional Hazards Regression Model Predicting Incident CKD by P-NfL Levels

Participants were re-examined for eGFR, 5.5 (5.3–6.1) years (median; IQR) after the first visit. A total of 558 individuals without CKD at baseline had re-examination data for eGFR (75% response rate, age 76 (76–77) years, 48% males, 76 new cases of CKD). Participants were stratified into quartiles by P-NfL levels from low to high, and the predictive value of P-NfL levels on incident CKD was estimated through a Cox proportional hazards model. P-NfL levels in the highest quartile were found to be a significant predictor of incident CKD after adjustment for age, sex, education, hypertension, diabetes (model II) and additional adjustments for smoking, BMI, fasting plasma glucose, LDL cholesterol, CRP, and homocysteine (model III) (Table 3). However, additional adjustment for baseline eGFR completely removed the longitudinal association.

**Table 3:**
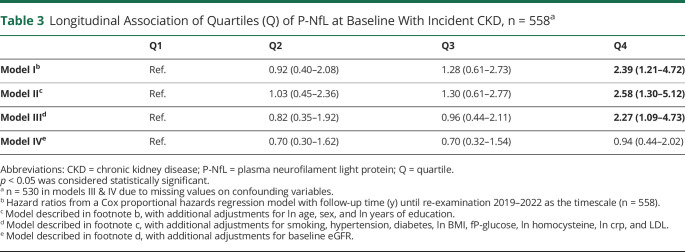
Longitudinal Association of Quartiles (Q) of P-NfL at Baseline With Incident CKD, n = 558^a^

### Stratification by Aβ Pathology

Participants with available CSF data were stratified based on Aβ status, and correlation analyses between fluid biomarkers of neurodegeneration and eGFR were performed (Table 4). In total, 142 (46.9%) of the 313 participants were classified as positive for Aβ pathology. In Aβ-positive participants, eGFR correlated with higher with Aβ42 (R = 0.25, *p* = 0.003), Aβ42/40 (R = 0.23, *p* = 0.004), and Aβ42/p-tau (R = 0.23, *p* = 0.005) and with lower t-tau levels (R = −0.16, *p* = 0.048). There was no significant correlation with p-tau, although the R coefficient and *p* values were similar to the eGFR/t-tau association (R = −0.15 and *p =* 0.071). In Aβ-negative participants, no correlations were seen for eGFR with any CSF biomarker. By contrast, eGFR correlated with lower P-NfL levels in both Aβ-positive and Aβ-negative participants with a similar R coefficient (R = −0.27 and R = −0.31, respectively) and *p* values (*p* < 0*.*001 for both correlations). The 5 participants with dementia and CSF data were excluded in a sensitivity analysis, without any significant influence on R coefficients or *p* values (eTable 5, links.lww.com/WNL/C849 in the Supplement). The correlation between t-tau and eGFR changed from *p* = 0.048 to *p* = 0.070 resulting in a shift over the *p* value threshold of 0.05, while R coefficients were still similar (R = −0.16 to R = −0.15).

**Table 4:**
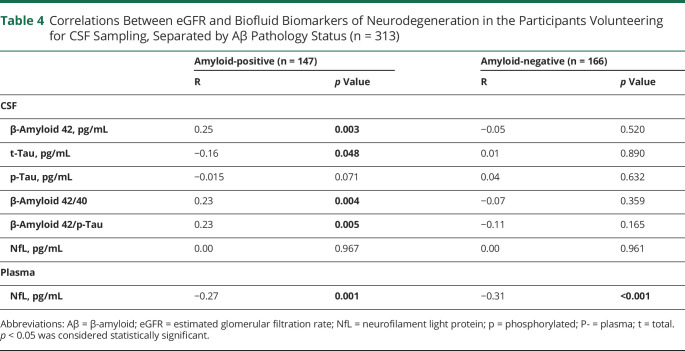
Correlations Between eGFR and Biofluid Biomarkers of Neurodegeneration in the Participants Volunteering for CSF Sampling, Separated by Aβ Pathology Status (n = 313)

## Discussion

In this study, we assessed several different markers of neurodegeneration in 744 individuals from a population-based cohort of 70-year-olds, in relation to the presence or absence of CKD. We found that P-NfL was higher in cognitively healthy individuals with CKD and presented similarly high levels as seen in participants with mild dementia. The association between CKD, eGFR, and P-NfL was still significant after adjustment for several confounding variables in a logistic regression model. P-NfL was an independent predictor of incident CKD in a Cox proportional hazards model. Furthermore, the linear regression coefficient for eGFR with P-NfL seemed steeper in participants with CKD compared with participants with normal kidney function. We found that CKD did not influence any MRI measurement of neurodegeneration, and no MRI variable correlated with eGFR. In a substudy including a smaller sample of 313 participants with CSF data, Alzheimer-related biomarkers only correlated with eGFR in Aβ42-positive participants after stratification for Aβ42 pathology.

CKD has previously been associated with markers in Alzheimer pathology,^5-7,9,18,42,43^ but because several risk factors and comorbidities are shared between the 2 conditions, it is difficult to determine the specific underlying mechanism. In this study, we did not observe any differences in structural MRI measurements between participants with or without CKD. Furthermore, we did not observe any difference in the established CSF biomarkers, between the 2 groups in the smaller sample of participants contributing with the CSF. The presence of CKD therefore did not seem to have any major confounding influence on CNS integrity in any aspect in this community-based sample. However, there was an association between P-NfL and both CKD and eGFR, indicating that kidney function is associated with some form of neurodegenerative pathology.^6,7,19^

The association between P-NfL and eGFR has been reported in several independent studies previously, including cognitively healthy Mexican Americans, non-Hispanic Whites, and participants from the Mayo Clinic Study of Aging.^8,12,18,44,45^ Studies in patients with diabetes and in children with congenital CKD have discussed potential links to NfL released from the CNS,^5,12^ while a study of patients with end-stage renal disease did not find any correlation between P-NfL and cognitive function measured through Mini-Mental State Examination.^46^ Support for a causal influence of CKD on neurodegeneration in CNS is found in children with congenital CKD, where P-NfL is elevated and cognitive impairment can be observed, possibly through shared genetic drivers.^5^ By contrast, a recent study including patients from French memory clinics did not find any influence of CKD status on the predictive value of circulating NfL on incident dementia.^47^ This could be explained by differences in study design and participant characteristics because the study was conducted in a clinical sample of patients with memory symptoms and measured longitudinal outcomes on incident dementia. Furthermore, P-NfL is elevated in polyneuropathy, a condition often seen in patients with CKD.^11,48^ Other community-based studies have previously reported observations on the influence of comorbidities on plasma-based biomarkers and reported an association between P-NfL and CKD.^6,7,19^ We extended these observations with measurements in a large Scandinavian community-based sample selected by birth date and found an association between CKD, eGFR, and P-NfL, using logistic and linear regression models adjusted for somatic comorbidities including diabetes and hypertension. We also found that P-NfL is a predictor of incident CKD. Furthermore, using stratified linear regression analysis, we observed that the association between P-NfL and eGFR is mainly manifest in participants with CKD. This indicates that the integrity of P-NfL as a marker to rule out underlying neurodegeneration in a general population should not be confounded to any larger degree in individuals with normal kidney function. While there was an association between P-NfL and eGFR, the levels of eGFR were similar between individuals with and without dementia in the range >60 mL/min/1.73 m^2^, suggesting that eGFR per se may not be considered an indicator of underlying neurodegenerative disease.

Memory impairment is sometimes caused by conditions that are not related to neurodegeneration for example, depression. It is therefore a medical challenge in primary care and in clinical interventions studies to segregate groups with these symptoms by underlying etiology. P-NfL has shown great promise in identifying individuals who present cognitive symptoms without any underlying neurodegenerative condition, providing support on which patients to refer to a memory clinic.^21^ It is also feasible for this purpose because it is a blood-based biomarker. Our observations of a similar elevation of P-NfL in individuals with CKD as in dementia indicate that P-NfL may not be useful for this purpose in individuals with CKD. Because our study is based on a community-based sample of older individuals, our participants are often found in the primary care setting.

We also measured CSF biomarkers in a smaller sample of individuals and did not find any influence of CKD status. While a previous study has shown that CSF NfL is associated with several other comorbidities, it does not seem to present any strong association with CKD.^49^ However, we observed a correlation between Aβ42, t-tau, and eGFR specifically in participants with Aβ pathology. CKD has previously been proposed to increase the risk of AD through several mechanisms, including increased levels of uremic toxins, calcium metabolism, and an altered hemodynamic regulation.^42^ Noteworthy, studies consistently propose a causal direction where CKD increases the risk of AD.^42^ In fact, CKD is one of the strongest risk factors of dementia.^50^ Considering previous reports, our results indicate that even a moderate decline in kidney function is associated with elevated markers of AD pathology. By contrast, the correlation between P-NfL and eGFR was independent of Aβ pathology status, indicating that the mechanisms previously discussed for P-NfL are not shared with the correlations to AD pathology.

There are some limitations to consider in this study. Almost all participants included in the study were born in Europe, and studies in other parts of the world may find different results due to variations in environment, life style, and genetics. This study does not provide any causal evidence regarding the association between NfL, CKD, and eGFR. In the longitudinal analysis, high P-NfL was associated with an increased risk of CKD. However, it should be considered that the Cox regression model was not significant after adjustment for baseline eGFR, and it is possible that the association found is mainly caused by a collinearity between eGFR and P-NfL. It is possible that the elevated levels of P-NfL are a consequence of impaired renal clearance, as proposed by others.^7^ However, the similarity in size between NfL (68 kDa^16^) and albumin (67 kDa) indicates that clearance should mainly be altered in patients with albuminuria and not all patients with CKD because proteins of this size do not normally pass the glomeruli. Studies with arterio-venous samples close to the kidney could provide the answer to this question. Regarding other limitations, the number of participants with dementia is low in this population-based sample and the results are not directly transferrable to clinical settings of patients with dementia. Furthermore, we did not have information on participant status of peripheral neuropathy, which limits the possibility to determine the main contributor of NfL in plasma. However, because there was no correlation between CSF-NfL and eGFR, it is also possible the periphery significantly contributes to plasma concentrations of NfL. Our observations on the correlation between CSF markers specific for AD and eGFR in participants with Aβ pathology were made in a limited sample and should be replicated in larger studies. Nonetheless, previous studies have reported similar observations, indicating that this observation in a population-based sample is valid.

Future longitudinal primary care studies are warranted to evaluate the added value of measuring P-NfL in detecting individuals with a higher specificity and predictive performance, which will be the basis for the modification of existing clinical practices. The precision of P-NfL as a predictor of neurodegeneration in individuals with CKD should also be determined in larger community-based and clinical studies. Although more studies are needed, it would be wise for clinicians to take CKD status into account when interpreting P-NfL measurements. The predictive role of P-NfL on incident CKD should also be validated in other settings because it could indicate that some neurodegenerative conditions may lead to an impaired renal function over time.

In a community-based cohort of 70-year olds, P-NfL was associated with both prevalent and incident CKD, while CSF and/or imaging measures did not differ by CKD status. Participants with CKD and dementia presented similar levels of P-NfL, which should be considered when using P-NfL as a neurodegeneration biomarker. Our observed correlations between Aβ42 and eGFR could indicate an association between kidney function and AD.

## Glossary

Aβ: β-amyloid
AD: Alzheimer disease
BMI: body mass index
CDR: clinical dementia rating
CKD: chronic kidney disease
COPD: chronic obstructive pulmonary disease
CRP: C-reactive protein
eGFR: estimated glomerular filtration rate
HDL: high-density lipoprotein
LDL: low-density lipoprotein
LP: lumbar puncture
NfL: neurofilament light protein
P: plasma
p: phosphorylated
t: total
